# Membrane and Films Based on Novel Crown-Containing Dyes as Promising Chemosensoring Materials

**DOI:** 10.3390/ma3125293

**Published:** 2010-12-17

**Authors:** Sergei Yu. Zaitsev, Daria O. Solovieva, Ilia S. Zaitsev

**Affiliations:** 1 Moscow State Academy of Veterinary Medicine and Biotechnology, Acad. Skryabina Str. 23, Moscow 109472, Russia; E-Mails: d.solovieva@mail.ru (D.O.S.); chemil@inbox.ru (I.S.Z.); 2 Institute of Bioorganic Chemistry, Russian Academy of Sciences, Miklucho-Maklaya Str. 16/10, Moscow 117871, Russia

**Keywords:** crown-containing dyes, monolayers, membranes, polymer films, ion-selectivity, photosensitivity, fluorescence

## Abstract

This paper discusses several works on supramolecular systems such as monolayer and multilayer, polymer films of various crown-containing dyes, surface-active monomers and polymers. Design, production and investigation of the membrane nanostructures based on crown ethers is a rapidly developing field at the “junction” of materials sciences and nanotechnology. These nanostructures can serve as convenient models for studying the self-organization and molecular recognition processes at interfaces that are typical for biomembranes. Based on the results obtained for such structures by absorption and fluorescence spectroscopy, atomic force and Brewster-angle microscopy, surface pressure and surface potential isotherm measurements, the possibility of developing micro- and nanomaterials possessing a set of specified properties (including chemosensor, photochromic and photorefractive materials) is demonstrated.

## 1. Introduction

Design and preparation of the ultrathin films based on crown-containing dyes (CCDs) is currently one of the most interesting and rapidly developing areas of research at the “junction” of polymers and colloids, physical-organic and biological chemistry, bio- and nanotechnology [1,2,3]. Such membrane nanostructures have already been used in various fields of chemistry, physics, biology, and medicine [3,4,5,6,7,8,9,10,11,12]. The latest achievements in this area are associated with the inception and fast development of a new sphere of interdisciplinary science—nanotechnology [1,2,3]. One possible definition of nanotechnology can be formulated as follows: a modern area of physicochemical and biomedical sciences that is concerned with studies of the methods for the production and operation of nanodimensional systems and application of the knowledge acquired to improve the existing (and to develop fundamentally new) technological processes and composite materials based on synthetic and natural compounds [3]. Such materials can be exemplified by the membrane systems widely used to simulate the structure and functions of biomembranes [2,10,11,12], namely, monomolecular layers (monolayers) and Langmuir-Blodgett (LB) films [4,5,6,7,12,13,14,15,16], flat bilayer lipid membranes [2,11,12,13], and spherical bi- and multilayer membranes (vesicles, liposomes, and their analogs) [5,6,11,12,13,14,16]. The principal shortcoming of such systems is their low stability, making them distinct from natural membranes stabilized by the electrostatic and hydrophobic interactions between integral and peripheral proteins, peptides, and glycolipids [5,6,7,8,9,12,16]. One of the first and most successful approaches to solving the problem of producing membrane nanosystems was achieved in the manufacture of polymer monolayers and liposomes on the basis of surface active monomers (SAMs) described by Fendler [5] and Bader and coworkers [6]. The following points are the most important for developing the theory and practice of producing function oriented membranes and nanostructures possessing a set of specified properties: (1) selecting the optimal ways to synthesize the SAMs of a specified structure, (2) optimizing the methods for obtaining the ultrathin organized films of these SAMs and establishing the self-association mechanism of mixed SAM and CCD mono and multilayers, (3) studying the reactions occurring in these layers in order to form certain nanostructures and developing physicochemical methods for investigating various types of ultrathin organized SAM and CCD base films, and (4) determining systems that are promising for subsequent use in the capacity of functional membranes and other types of nanomaterials.

## 2. Chemosensoring Monolayers for Detection of Some Alkaline and Alkaline-Earth Metal Cations 

**Figure 1:**
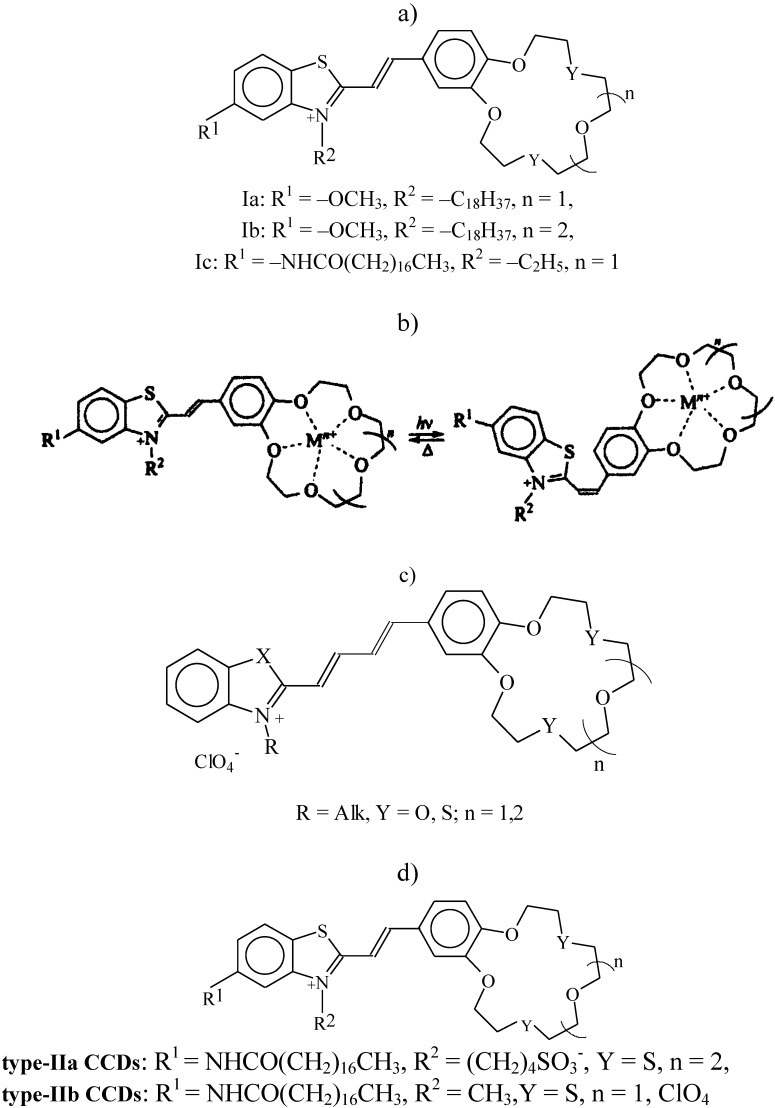
(**a**) Structure of type-I crown-containing styryle dyes (CCDs); (**b**) schematic diagram of the reversible *trans-cis* isomerization of crown ether molecule; (**c**) structure of butadienyl derivatives of crown ethers (BDCE); and (**d**) structure of type-II crown-containing styryle dyes (CCD).

The properties of a new class of photochromic materials synthesized at the Photochemistry Center of the Russian Academy of Sciences [17], such as crown-containing styryl dyes (type I CCD, Figure 1a), have recently become the subject of intense studies. The presence of a crown ether fragment in crown-containing dyes facilitates their selective bonding to metal cations. Spectral measurements taken in organic and water-organic media have shown cation influences the physicochemical propertied of the sensor molecule [17]. The photochromic properties of CCDs are the reason they can transform during two photoinduced reversible reactions (Figure 1b), namely, *trans-cis* C=C double-bond isomerization and [2 + 2] photocycloaddition to form substituted cyclobutanes [17].

**Figure 2:**
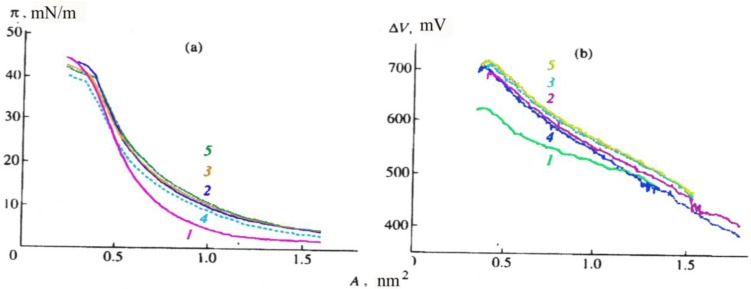
(**a**) Surface pressure (π), and (**b**) surface potential (∆V), as a function of the surface area (A) per molecule of a type-Ib CCD in a monolayer of the surface of(1) distilled water and 1 mM solutions of (2) CaCl_2_, (3) MgCl_2_, (4) KCl, and (5) NaCl.

Studies of CCDs types Ia–Ic [18,19,20,21] showed them to be capable of forming stable monolayers at the air-water and water–alkali metal salt solution interfaces (Figure 2, the isotherms presented refer to CCDs type Ib). Their main parameters are as follows: surface area (A) per CCD molecule, surface pressure (π), and potential (ΔV); the collapse pressure and potential of the monolayer are at their minimum at the water surface and grow higher in the presence of salts in the water subphase (Figure 2a and Figure 2b), which points to an interaction between CCDs and alkaline and alkaline earth metal cations. Some authors [18,19,20,21] believe that the inclusion of a cation in a CCD monolayer increases the mutual repulsion of the positively charged molecules and the surface area (A) per type-Ib CCD molecule in the monolayer (Figure 2a).

**Figure 3:**
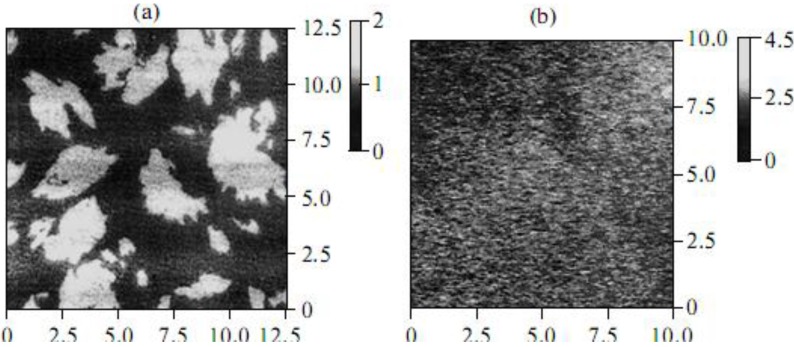
Atomic force micrographs of type-Ib CCD monolyers transferred by the Langmuir-Blodgett method onto a mica substrate from the surface of (**a**) water and (**b**) a 1 mM NaCl solution. In both panels, on the left is the structure of the object; on the right is the light scale characterizing the height of the objects in nm.

That the area occupied by a type-Ic CCD molecule on the surface of water and 1 mM salt solutions was found to be half of that for type-Ia and type-Ib CCD molecules points to a pronounced change in the molecular organization of the monolayer as a result of the translocation of the hydrophobic aliphatic substitute from position 3 to position 5 in the benzothiazole ring structure [19]. Based on the data obtained by the methods of the surface-enhanced Raman scattering (SERS) and Brewster-angle microscopy (BAM) [19,20], it was concluded that the structures of CCD monolayers formed on the surface of water and various salt solutions differed substantially. To study the structure of CCD monolayers in more detail, atomic force microscopy (AFM) was used. The analysis of AFM data showed that a type-Ib CCD monolayer transferred by the Langmuir-Blodgett method from the surface of water onto mica is an accumulation of a large number of aggregates differing in size (light areas in Figure 3a).

It is important that the height of the aggregates is 1.8 nm on average, which corresponds to the length of the aliphatic substitute in type-Ib CCDs. In the presence of salts (for example, NaCl), a “practically homogeneous” type-Ib CCD monolayer forms (Figure 3b). Such changes in the structure of monolayers are also characteristic of other CCDs, which is additional evidence of the interaction between the dye in the monolayer and cations in the water subphase.

**Figure 4:**
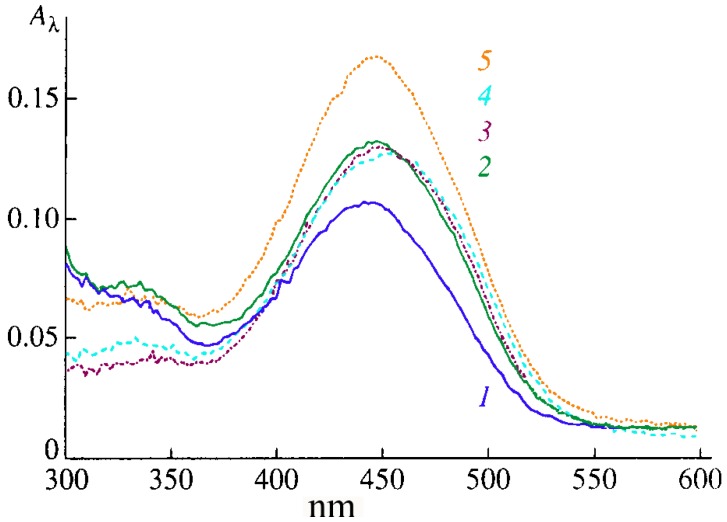
Absorption spectra of type-Ib CCDs in monolayers on the surface of: (1) distilled water and 10 mM solutions of (2) KCl, (3) CaCl_2_, (4) MgCl_2_, and (5) NaCl at 10 mN/m.

Valuable information on the packing of CCDs in monolayers directly at interfaces was obtained from absorption and fluorescence spectroscopy data [19,20,21,22]. For example, an intense absorption band is present in the electronic spectra of types Ia–Ic CCD monolayers (recorded directly on the surface of distilled water and 10 mM salt solutions) in the region of 450 ± 5 nm (Figure 4). As the surface pressure π is increased from 5 to 30 mN/m, the absorption maximum in the spectrum of type-Ib CCD monolayers is observed to shift from 446 to 438 nm, which is due to the formation of the aggregates detected earlier by the AFM method. The increase in absorption is associated with the surface density of chromophores growing higher upon the contraction of the CCD monolayer. The maximum absorption intensity in the spectra of type-Ib monolayers in the presence of NaCl substantially exceeds the absorption intensity of similar monolayers in the presence of CaCl_2_, MgCl_2_, or KCl (Figure 4), which is due to the selectivity of cations bonding to the CCD in the monolayer [19,20,21].

**Figure 5:**
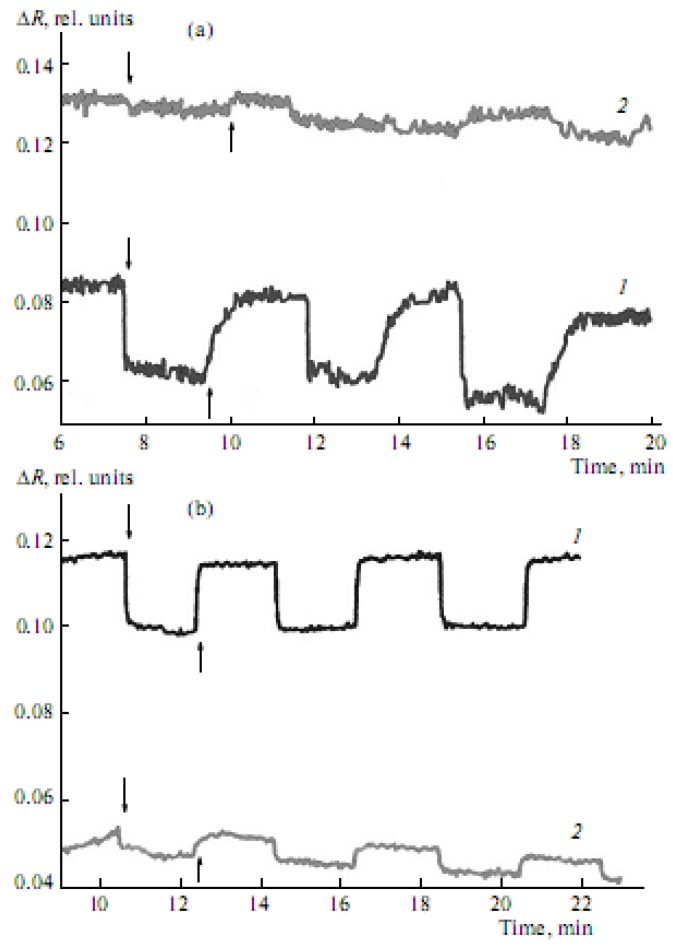
Reversible changes in the intensity of absorption at λ = 446 nm of type-Ib CCDs as a function of the time of exposure to light with a wavelength of 438 nm and dark relaxation of the monolayers formed on the surface of (**a**) 10 mM KCl solution at surface pressures of (1) 3 mN/m and (2) 9 mN/m, and (**b**) 10 mM NaCl solution at surface pressures of (1) 3 mN/m and (2)14mN/m (↓—indicates that the light is on; ↑—indicates that the light is off).

It is important that the exposure of type Ia–Ic CCD monolayers, formed on the surface of salt subphases, to light at constant π is observed to cause reversible changes in their absorption intensity (Figure 5a, Figure 5b). The absorption intensity (at 446 nm) of type Ia CCD monolayers is, in this case, observed to decrease sharply a few seconds after their photoactivation with light OF 438 nm in wavelength, which is close to the absorption maximum of the CCD. Once the absorption intensity becomes constant, irradiation is stopped and the absorption intensity (at 446 nm) starts increasing until it almost reaches its original values (Figure 5a and Figure 5b).

These reversible changes in the absorption intensity are associated with the *trans-cis* and *cis-trans* C=C double-bond isomerization processes in the crown ether molecule (Figure 1b). These effects are characteristic of other amphiphilic dyes as well [17,19]. The nature of the metal cation and the character of molecule organization in the monolayer influence the photoisomerization process. At low surface pressures (around 3 mN/m), the reversible changes in the absorption intensity of type-Ib monolayers formed on the surface of a 10-mM KCl solution (Figure 5a, curve 1) are somewhat greater than those of similar monolayers obtained on the surface of a 10 mM NaCl solution (Figure 5b, curve 1) under similar conditions. However, at higher surface pressure values (about 9 mN/m), these changes for type-Ib CCD monolayers on the surface of a 10 mM KCl solution (Figure 5a, curve 2) are substantially smaller than those for similar monolayers on the surface of a 10 mM NaCl solution (Figure 5b, curve 2); this is so even at still higher surface pressures (around 14 mN/m). Thus, optimal conditions for forming stable monolayers and their interaction with alkaline and alkaline-earth metal cations were found for types Ia–Ic CCDs differing in cycle size, as well as in the length and position of the alkyl substitute in the molecule.

## 3. Chemosensoring Monolayers for Detection of Some Heavy Metal Cations

**Figure 6:**
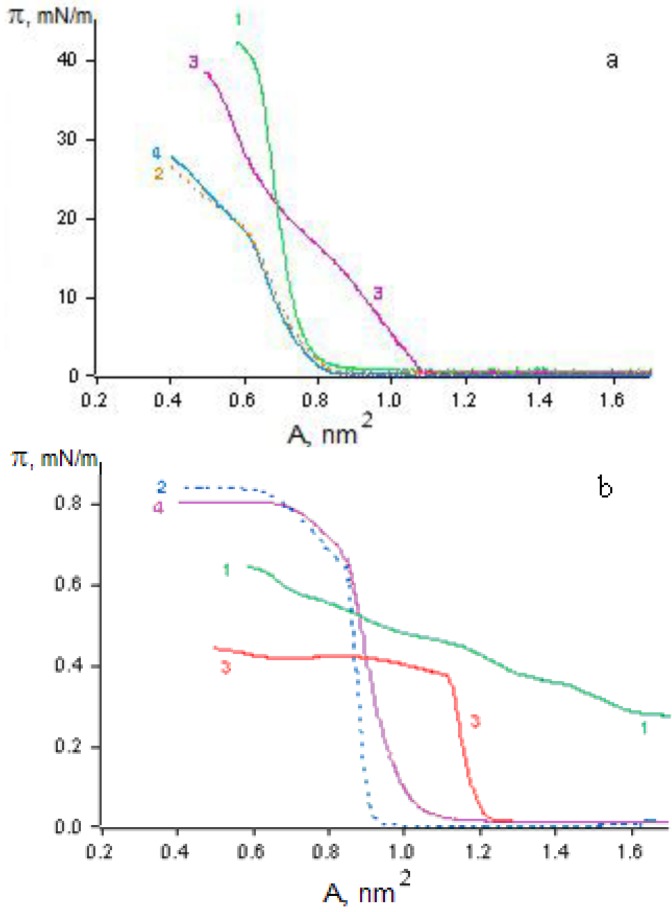
(**a**) Surface pressure (π); and (**b**) surface potential (∆V), as a function of the surface area (A) per butadienyl derivatives of crown ethers (BDCE) molecule in monolayers formed on the surface of (1) distilled water, and 1 mM solutions of (2) KClO_4_, (3) Hg(ClO_4_)_2_, and (4) Pb(ClO_4_)_2_.

Researchers at the Photochemistry Center of the Russian Academy of Sciences synthesized a new amphiphilic butadienyl crown ether (BDCE) (Figure 1c) with two sulfur atoms in the crown-ether ring-structure of the molecule [23,24]. BDCE was found to be capable of forming relatively stable monolayers on the surface of water (with a collapse pressure around 42 mN/m), as well as solutions of alkaline salts (π = 40–45 mN/m) and heavy metal salts (π = 23–40 mN/m), which in most cases is substantially higher than the collapse pressure for the monolayers of the dithio derivatives of the CCDs (π = 25.5–34.5 mN/m) studied by us earlier [19,20,21]. The material difference between the isotherms of BDCE monolayers on the surface of 1 mM Hg(ClO_4_)_2_ solution and those of similar monolayers on the surface of other salt solutions, as well as water (Figure 6a), bears witness to a specific interaction between the BDCE macrocycle and the Hg^2+^ cation. This explains the substantial change occurring on the surface area per BDCE molecule in the presence of Hg^2+^ cations (Figure 6a, curve 3) in comparison with that in the presence of other heavy metal cations (Figure 6a, curves 2 and 4) and water (Figure 6a, curve 1). The potential ΔV for BDCE monolayers on the surface of water gradually grows higher (380 mV rise) as the monolayer surface area decreases (Figure 6b, curve 1), which points to a gradual “uplift” of the BDCE chromophores upon the contraction of the monolayer. On the contrary, a “stepwise” increase of ΔV by 400 mV is observed in the case of BDCE monolayers on the surface of a 1 mM Hg(ClO_4_)_2_ solution (Figure 6b, curve 3), which differs from the change of ΔV (a sharp increase by 800 mV) for similar monolayers on the surface of 1 mM solutions of KClO_4_ and Pb(ClO_4_)_2_ (Figure 6b, curves 2 and 4). This is additional proof of the specific interaction between BDCE and Hg^2+^ cations.

**Figure 7:**
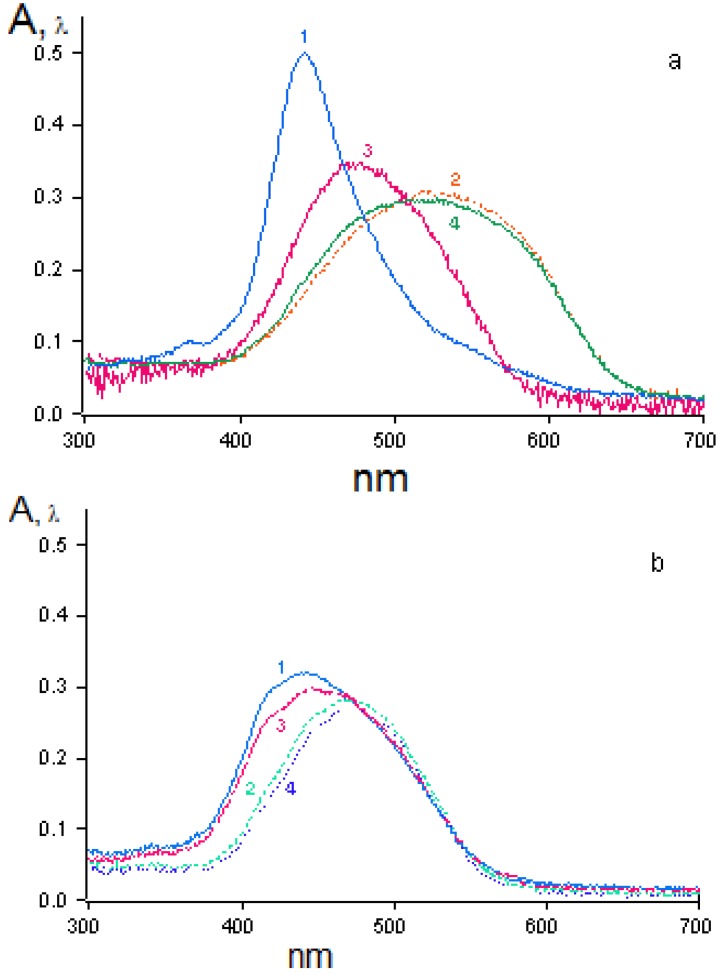
Absorption spectra of BDCE in monolayers on the surface of (**a**) distilled water, and (**b**) 1 mM Hg(ClO_4_)_2_ solutions at pressures of (1) 30 mN/m; (2) 20 mN/m; (3) 10 mN/m; and (4) 1 mN/m.

An absorption band in the visible region of the spectrum can be detected in the spectra of BDCE monolayers on the surface of all the subphases studied. For example, the absorption maximum of the monolayers on the surface of water is at 442 nm (Figure 7a, curve 1). The absorption spectra of the monolayers on the surface of 1 mM KClO_4_ and Pb(ClO_4_)_2_ solutions differ substantially from those of the monolayers on water; their absorption maxima is in the region of 500–600 nm (Figure 7a, curves 2 and 4). The absorption spectra of the monolayers on the surface of a 1 mM AgClO_4_ solution (Figure 7a, curve 3) differ from those of the monolayers on the surface of 1 mM KClO_4_ and Pb(ClO_4_)_2_ solutions, both quantitatively (the absorption maximum is at 474 nm) and qualitatively. These effects are due to the differences in the type of molecular structures present in the monolayers. One is the monomer form of the dye, the second (with the absorption maximum shifted toward longer wavelengths) is characterized by the formation of “large” BDCE aggregates, and the third (with the absorption maximum shifted toward shorter wavelengths) is characterized by the formation of BDCE“dimers” [25,26,27]. In the case of 1 mM Hg(ClO_4_)_2_ solution, the absorption spectrum of the monolayer at π = 1 mN/m has a broad band with the maximum at 470 nm (Figure 7b, curve 4), which is shifted to 442 nm upon contraction of the monolayer at π = 30 mN/m (Figure 7b, curve 1).

There is more proof of the specific interaction between the BDCE and Hg^2+^ cation [24,25,26]. Small BDCE domains were found by the Brewster-angle microscopy method in the monolayers on the surface of water and 1 mM KClO_4_ and Pb(ClO_4_)_2_ solutions. In contrast, BDCE monolayers on the surface of the Hg(ClO_4_)_2_ solution are practically homogeneous, which is a peculiar visualization of the specific interaction between BDCE and Hg^2+^. What is more, it was only with the BDCE monolayers on the surface of the mercury perchlorate solution that reversible changes in reflected intensity were observed to occur upon the photo-activation of the dye, which is of great importance in the development of future nanomaterials with desired properties [27,28]. Interesting results were obtained when studying the monolayers of surface active derivatives of 18-(15)-dithiacrown-6(5) (a type-II CCD, Figure 1d) also synthesized at the Photochemistry Center of the Russian Academy of Sciences [17,21]. The development of monolayers of these CCDs mixed with surface-active monomers or polymers yields nanosystems whose properties can be controlled as desired by varying the orientation and packing of molecules in the monolayers, as well as their photosensitivity and ion selectivity, and improving their stability and strength. That is confirmed by the results of investigations of the monolayer properties of type-II CCDs mixed with various surface-active monomers and polymers in the range of proportions from 1:10 to 10:1 formed on the surface of water and solutions of various salts [21,23,24,25,26]. For monolayers of type-IIa or type-IIb CCDs mixed in equimolar ratios with poly(16-di(metacryloyloxyhexadecanoyl)glicero-3-phosphoryl choline (16-MHPC), no “deviations from additivity” are observed at 20 mN/m on the surface of water. In contrast, on the surface of mercury perchlorate solutions, “negative deviations from additivity” (by 22%) are observed in the case of type-IIa CCDs and “positive deviations from additivity” (by 49%) are seen in the case of type-IIb CCDs. These effects are due to the specific interaction between type-II CCDs and mercury cations from the water subphase. Thus, crown-containing dyes in organized nanostructures with polymers exhibit a capacity for specific interactions with metal cations and the controllable alteration of the properties of these nanostructures [27,28].

## 4. Polymer Films for Detection of Some Heavy Metal Cations

**Figure 8:**
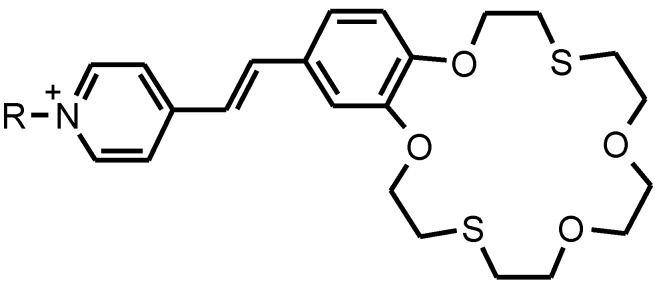
The structure of the amphiphilic pyridinium benzodithia-18-crown-6 dye (**1**).

There are three functional parts in the structure of the amphiphilic pyridinium benzodithia-18-crown-6 dye (**1**) (Figure 8): (a) an ion-selective part of the dithia-containing macrocycle, (b) a photosensitive benzo-C=C-pyridinium part, and (c) a lipid-like hydrophobic “tail” (C18) prepared by *N*-alkylation of 4-methylpyridine with 1-bromooctadecane. The details of the synthesis and physical-chemical characterization of the dye **1** has been previously described [29].

The polymer films with the dye **1** (total thickness of 40 ± 5 µm) were prepared by modified spreading technique [30,31] on the medical glass substrate (30 × 10 mm) from 10% polymer solutions in chloroform. Numerous experiments showed that this dye did not dissolve in the aqueous solutions and was not extracted from the polymer films by interaction with studied salts. The spectra of the polymer-dye films were recorded in the range 300–800 nm using absorption and fluorescence spectrophotometers. The absorbance of the polymer films without dye was below 300 nm. The influence of heavy cations on these films was studied using 10^−3^, 10^−4^, 10^−5^ and 10^−6 M Hg(ClO^_4_)_2_ or AgClO_4_ aqueous solutions. The spectra of the dye-polymer films in all cases showed a strong band with an absorption maximum at 400–450 nm, which was assigned to the dye *trans*-form according to published data [3,31,32,33,34] on the pure dye in some organic solvents. The pronounced shoulder at 320–350 nm was assigned to the dye *cis*-form according to our preliminary studies [3].

Applying various salts and their concentrations to the polymer-dye film, it was possible to directly obtain changes in the dye **1** spectral parameters (the wavelength maximum and its shift). For example, the absorption wavelength maximum for the polymer-dye (thickness 10 mm) film based on PVC was at 419 nm and absorption intensity about 0.160 arbitrary units. The maximal shifts of absorption on about −26 nm for the polymer-dye film were found in the case of 10^−3^ M Hg(ClO_4_)_2_ aqueous solutions (Table 1).

**Table 1:** The wavelength for the absorption maximum (nm) and absorption intensity (a.u.) for the polymer-dye film based on PVC before (λ_0_, I_0_) and after (λ, I) treatment with Hg(ClO_4_)_2_ solutions.

Serial number	λ_0_, nm	I_0,_ a.u. (before)	Hg^2+^ M	λ, nm	I, a.u. (after)	Δλ, nm shift
1	419 ± 1	0.16 ± 0.04	10^−3^	393 ± 1	0.18 ± 0.04	−26
2	419 ± 1	0.26 ± 0.04	10^−4^	416 ± 1	0.24 ± 0.04	−3
3	419 ± 1	0.17 ± 0.04	10^−5^	415 ± 1	0.18 ± 0.04	−4
4	419 ± 1	0.16 ± 0.04	10^−6^	418 ± 1	0.18 ± 0.04	−1

There were no linear dependences between salt concentration in solution, and the wavelength shift for the absorption maximum was found in the case of Hg(ClO_4_)_2_ but, in general, such a system and method can be used for the detection of mercury cations at 1 mM concentrations in aqueous solutions.

The same polymer-dye films based on PVC were used for detection of silver cations in aqueous solutions and showed even better results. The maximal shifts of absorption on about −25 nm for the polymer-dye film were found in the case of 10^−3^ M AgClO_4_ aqueous solution (Table 2).

**Table 2:** The wavelength for the absorption maximum (nm) and absorption intensity (a.u.) for the polymer-dye film based on PVC before (λ_0_, I_0_) and after (λ, I) treatment with AgClO_4_ solutions.

Serial number	λ_0_, nm	I_0,_ a.u. (before)	Ag^+^ M	λ, nm	I, a.u. (after)	Δλ, nm shift
1	419 ± 1	0.17 ± 0.04	10^−3^	394 ± 1	0.18 ± 0.04	−25
2	419 ± 1	0.26 ± 0.04	10^−4^	405 ± 1	0.23 ± 0.04	−14
3	419 ± 1	0.15 ± 0.04	10^−5^	412 ± 1	0.16 ± 0.04	−7
4	419 ± 1	0.16 ± 0.04	10^−6^	418 ± 1	0,17 ± 0,04	−1

Linear dependence between salt concentration in solution and the wavelength shift for the absorption maximum was found in the case of AgClO_4_ solutions, as compared to the Hg(ClO_4_)_2_ solutions, which could be promising for the quantitative detection of the silver cations in aqueous solutions.

The fluorescence wavelength maximum for the polymer-dye (thickness 10 mm) film based on PVC was at 502 nm and fluorescence intensity about 450 arbitrary units. The maximal shifts of fluorescence on about −15 nm for the polymer-dye film were found in the case of 10^−3^ M Hg(ClO_4_)_2_ aqueous solutions (Table 3).

**Table 3:** The wavelength for the fluorescence maximum (nm) and absorption intensity (a.u.) for the polymer-dye film based on PVC before (λ_0_, I_0_) and after (λ, I) treatment with Hg(ClO_4_)_2_ solutions.

Serial number	λ_0_, nm	I_0,_ a.u. (before)	Hg^2+^ M	λ, nm	I, a.u. (after)	Δλ, nm shift
1	502 ± 1	401 ± 1	10^−3^	487 ± 1	335 ± 1	−15
2	502 ± 1	672 ± 1	10^−4^	492 ± 1	313 ± 1	−10
3	502 ± 1	457 ± 1	10^−5^	500 ± 1	342 ± 1	−2
4	502 ± 1	659 ± 1	10^−6^	501 ± 1	333 ± 1	−1

It is important to underline the almost linear dependence between salt concentration in solution and the wavelength shift for the fluorescence maximum in the case of Hg(ClO_4_)_2_ solutions as compared to absorption maximum for those solutions. This could be promising for the quantitative detection of the mercury cations in aqueous solutions.

On the other hand, the maximal shifts of fluorescence on about −19 nm for the polymer-dye film were found in the case of 10^−3^ M AgClO_4_ aqueous solutions (Table 4).

**Table 4:** The wavelength for the fluorescence maximum (nm) and absorption intensity (a.u.) for the polymer-dye film based on PVC before (λ_0_, I_0_) and after (λ, I) treatment with AgClO_4_ solutions.

Serial number	λ_0_, nm	I_0_, a.u. (before)	Ag^+^ M	λ, nm	I, a.u. (after)	Δλ, nm shift
1	502 ± 1	456 ± 1	10^−3^	483 ± 1	345 ± 1	−19
2	502 ± 1	625 ± 1	10^−4^	506 ± 1	583 ± 1	+4
3	502 ± 1	500 ± 1	10^−5^	503 ± 1	458 ± 1	+1
4	502 ± 1	612 ± 1	10^−6^	503 ± 1	574 ± 1	+1

There were no linear dependences between salt concentration in solution and the wavelength shift for the fluorescence maximum found in the case of AgClO_4_ solutions as compared to the absorption maximum for these solutions. Therefore, this system is not very useful for the quantitative detection of silver cations in aqueous solutions.

Thus, the wavelength shift for the absorption maximum in the case of AgClO_4_ solutions and wavelength shift for the fluorescence maximum in the case of Hg(ClO_4_)_2_could indicate the most promising materials for novel optical chemosensor for quantitative detection of the silver and mercury cations in aqueous solutions.

## 5. Monolayers with Novel Photosensitive Bis-Crown-Ether Derivative for Detection of Small Organic Molecules 

As an example, the preparation of monolayers of the photosensitive bis-crown-ether derivative as an optical molecular sensor (OMS-5) and investigation of its properties both at the water-air interface and transferred onto optically transparent plates is discussed.

**Figure 9:**
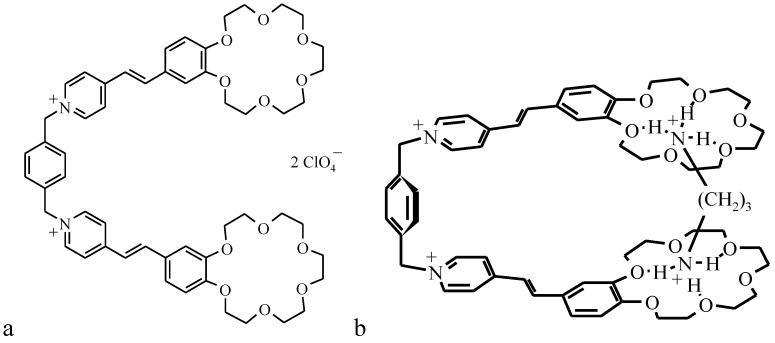
Structure of OMS-5 (**a**) and scheme of the 1:1 complex between OMS-5 and 1,3-propanediammonium ion (**b**).

The synthesis of OMS-5 (general structure shown in Figure 9a) was published recently [35,36].

For studying the OMS chemosensor properties, a few 1,ω-diaminoalkane dihydrochlorides [NH_3_^+^ (CH_2_)nNH_3_^+^ 2 Cl^−^ (n = 3 or 7)] and tetramethylammonium chloride (NMe_4_Cl) were used. The mixed monolayers (OMS-5: stearic acid = 1:2) were transferred onto quartz substrates by the Langmuir-Blodgett method at a constant surface pressure of 30 mN/m.

**Figure 10:**
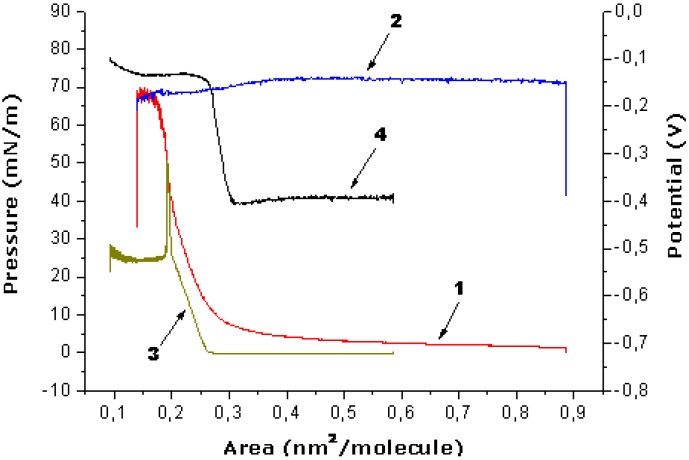
Dependence of surface pressure *vs.* monolayer area (1, 3) and surface potential *vs*. monolayer area (2, 4) for the mixture of OMS-5:C18 = 1:2 (1, 2) or pure stearic acid (3, 4) at the subphase of 1 mM solution of 1,3-diaminopropane dihydrochloride.

The novel OMS-5, which does not form stable monolayers itself, can be stabilized in the mixture with stearic acid (C18) at surfaces of various aqueous solutions (NMe_4_Cl, 1,3-diaminopropane and 1,7-diaminoheptane dihydrochlorides) with collapse pressures of 60–66 mN/m (Figure 10). It is important that the area increase in the mixed monolayer as compared to the pure stearic acid is directly proportional to the molar fraction of the OMS-5 in the mixture. The ratio of OMS-5 to stearic acid of 1:2 in the mixed monolayers was selected for the further study. The relative increases in the molecular area values for these mixed monolayers were 12% for 1,3-diaminopropane dihydrochloride or 4 % for NMe_4_Cl at the interface of the 1 mM solutions, as compared to distilled water (the area per stearic acid molecules in the mixed monolayer with OMS-5 was 0.25 nm^2^) at a constant surface pressure of 10 mN/m. The relative molecular area increase was about 16% for the mixed monolayers of OMS-5:C18 = 1:2 (Figure 10, curve 1) as compared to pure stearic acid on the same 1 mM solution of 1,3-diaminopropane dihydrochloride (Figure 10, curve 3).

The surface potential values (ΔV) for the mixed OMS-5:C18 monolayers at the interfaces of 1,3-diaminopropane dihydrochloride (Figure 10, curve 2) jumped up upon spreading (by about 0.20–0.25 V) and remained almost constant by monolayer compression in the whole studied areas range (from 0.85 to 0.20 nm^2^/molecule). In contrast, for pure stearic acid monolayers on the same 1 mM solution of 1,3-diaminopropane dihydrochloride, the surface potential values (Figure 10, curve 4) increase sharply (by about 0.25 V) only in the narrow area range (from 0.32 to 0.26 nm^2^/molecule) by monolayer compression just before the pronounced increase in surface pressure (Figure 10, curve 3). The isotherms of the prepared mixed monolayer are almost the same after expansion and second compression (after 2–3 hours), proving the stability of these obtained mixed “nanofilms”. The most complete transfer of these mixed monolayer onto quartz plates by the Langmuir-Blodgett method was found at constant surface pressure of 30 mN/m in the case of the 1 mM salt solutions.

**Figure 11:**
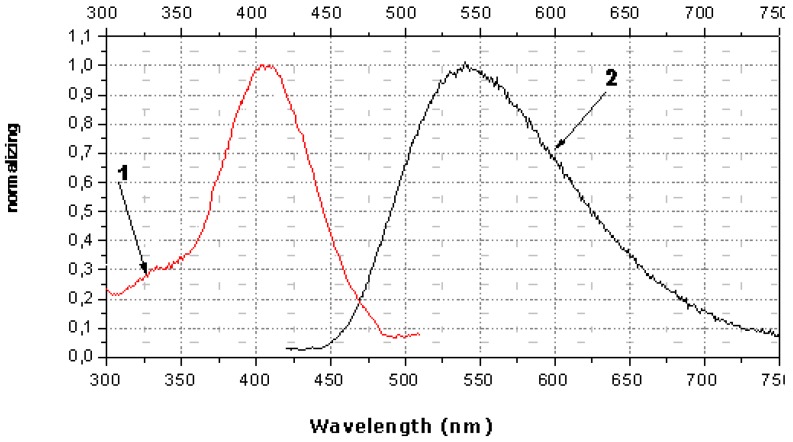
Spectra of absorption (curve 1, red) in comparison to fluorescence (curve 2, black) of mixed monolayer (OMS-5:C18 = 1:2) transferred from 1 mM solution of 1,3-diaminopropane dihydrochloride.

The spectral characteristics of OMS-5 in monolayers show the presence of a strong maximum of absorption (at 398 nm) and fluorescence (at 532 nm) for OMS-5 in solution and its mixture with C18 (both at the air-water interface and transferred onto quartz plates). A shift of the maximum of absorption to 394 nm and of fluorescence to 542 nm was observed for the mixed OMS-5:C18 monolayer transferred from the 1 mM solution of 1,7-diaminoheptane dihydrochloride. Moreover, a shift of maximum of absorption to 406 nm and fluorescence to 540 nm was found for this monolayer transferred from the 1 mM solution of 1,3-diaminopropane dihydrochloride (Figure 11). It is interesting that the shifts of the absorption maximum for these diammonium compounds were in the opposite direction as compared to the initial absorption maximum (at 398 nm) and the total difference was about 12 nm. These changes are connected with the formation of the complexes of various types between OMS-5 and particular diamines. The negligibly small shifts of absorption and fluorescence maxima were found for the mixed OMS-5:C18 monolayer transferred from the 1 mM solutions of tetramethylammonium chloride that can be due to the non-specific interactions between all components at the interfaces [36].

The maximal changes were found for the mixed OMS-5:C18 monolayer transferred from the 1 mM solution of 1,3-diaminopropane dihydrochloride due to the strong interaction between OMS-5 and this diammonium ion (having the sterically most appropriate spacer for interactions of positively charged ammonium groups with two crown-ether rings of OMS-5) forming a pseudocyclic 1:1 complex (Figure 9b). Such complex formation between OMS-5 and particular alkanediammonium salts can be promising for design of chemosensoring nanomaterials, capable for optical detection of biogenic diamines in solutions.

The interesting examples of the ditopic receptors for α,ω-alkanediyldiammonium cations based on a tetraazamacrocyclic (cyclidene) nickel(II) complex bearing two crown-ether residues have been discussed by coworkers of the Tufts University, USA [37]. The studies of the host-guest interaction between the receptors and a series of α,ω-diammonium salts showed that 1:1 complexes are formed, in agreement with our results. In particular, receptor with benzo-15-crown-5 arms showed substantial selectivity in binding of trimethylene- and tetramethylenediammonium dications, and 1–2 orders of magnitude weaker binding of shorter (C_2_) or longer (C_5_ and C_6_) diammonium cations [37].

## 6. Conclusions

In conclusion, Langmuir monolayers and thin polymer films based on crown-containing dyes are not only unique models for the fundamental studies of molecular organization and recognition phenomena, but also prototype of chemosensoring materials on various “small compounds” (such as organic and inorganic molecules and ions) with optical signal detection. On the other hand, the numerous works concerning self-assembled monolayers (as an alternative to Langmuir monolayers) of macrocyclic complexes with inorganic and organic molecules and ions have been published only during this year by various research groups worldwide [38,39,40,41]. In spite of the fact that these crown-containing compounds are not dye derivatives, they can serve as another important example for application of macrocyclic complexes as building blocks for molecular devices [42].
